# Phorbol esters dPPA/dPA promote furin expression involving transcription factor CEBPβ in neuronal cells

**DOI:** 10.18632/oncotarget.18569

**Published:** 2017-06-19

**Authors:** Jing-Si Zha, Bing-Lin Zhu, Lu Liu, Yu-Jie Lai, Yan Long, Xiao-Tong Hu, Xiao-Juan Deng, Xue-Feng Wang, Zhen Yan, Guo-Jun Chen

**Affiliations:** ^1^ Department of Neurology, The First Affiliated Hospital of Chongqing Medical University, Chongqing Key Laboratory of Neurology, Chongqing 400016, China; ^2^ Department of Physiology and Biophysics, State University of New York at Buffalo, Buffalo, NY, 14214, USA

**Keywords:** furin, dPPA/dPA, CEBPβ, ERK, PI3K

## Abstract

Using high-throughput small molecule screening targeting furin gene, we identified that phorbol esters dPPA (12-Deoxyphorbol 13-phenylacetate 20-acetate) and dPA (12-Deoxyphorbol 13-acetate) significantly increased furin protein and mRNA expression in SH-SY5Y cells. This effect was prevented by PKC (protein kinase C) inhibitor calphostin C but not Ro318220, suggesting that the C1 domain, rather than the catalytic domain of PKC plays an important role. Luciferase assay revealed that nucleotides −7925 to −7426 were sufficient to mediate dPPA/dPA enhancement of *furin* P1 promoter activity. RNA interference of transcriptional factors *CEBPβ* (CCAAT/enhancer-binding protein β) and *GATA1* revealed that knockdown of *CEBPβ* significantly attenuated the effect of dPPA on furin expression. Pharmacological inhibition of ERK and PI3K but not TGFβ receptor diminished the up-regulation of furin by dPPA. These results suggested that in neuronal cells, transcriptional activation of furin by dPPA/dPA may be initiated by C1 domain containing proteins including PKC; the intracellular signaling involves ERK and PI3K and transcription factor CEBPβ.

## INTRODUCTION

Furin is one of the proprotein convertases (PCs) [1], which are involved in the proteolysis of precursor proteins, including growth factors and hormones, receptors, and matrix metalloproteinases [2, 3]. Furin knockout mice are unable to survive due to cardiac developmental defects at 10.5 embryonic days [4]. Evidence has suggested that furin plays important role in cancer and infectious diseases [4, 5].

In the nervous system, furin promotes differentiation and collateral formation of nematode sensory neurons [6, 7]. The wide spectrum of substrates suggests that furin may be important in neuronal functions. For instance, furin catalyzes pro-BDNF (brain-derived neurotrophic factor) and pro-NGF (nerve growth factor) into corresponding mature forms [8, 9]. While mature BDNF/NGF plays critical role in the development and survival of neurons [10, 11], pro-BDNF inhibits synaptic plasticity and pro-NGF promotes apoptosis [12, 13]. Dysfunction of BDNF is associated with depression, schizophrenia, cerebral trauma and Alzheimer's disease (AD) [14]. Another important substrate of furin is pro-ADAM10 (A disintegrin and metalloproteinase domain-containing protein 10), the α-secretase that is closely associated with AD pathology [15, 16]. Interestingly, furin protein levels are significantly decreased in the brain of AD patients and animal models [17]. It is unclear how furin is regulated in neuronal cells.

Using high-throughput small molecules screening, we found that phorbol esters PMA (phorbol 12-myristate 13-acetate), PDBu (phorbol 12, 13-dibutyrate), dPA (12-deoxyphorbol 13-acetate) and dPPA (12-deoxyphorbol 13-phenylacetate 20-acetate), significantly increased *furin* luciferase activity. In this study, we found that dPPA and dPA that are not carcinogenic, could increase the expression of furin in neuronal cells. This effect was prevented by PKC inhibitor calphostin C. We further showed that transcription factor CEBPβ and ERK/PI3K signaling pathways were involved in this regulation.

## RESULTS

### dPPA/dPA promoted furin expression

**Figure 1:**
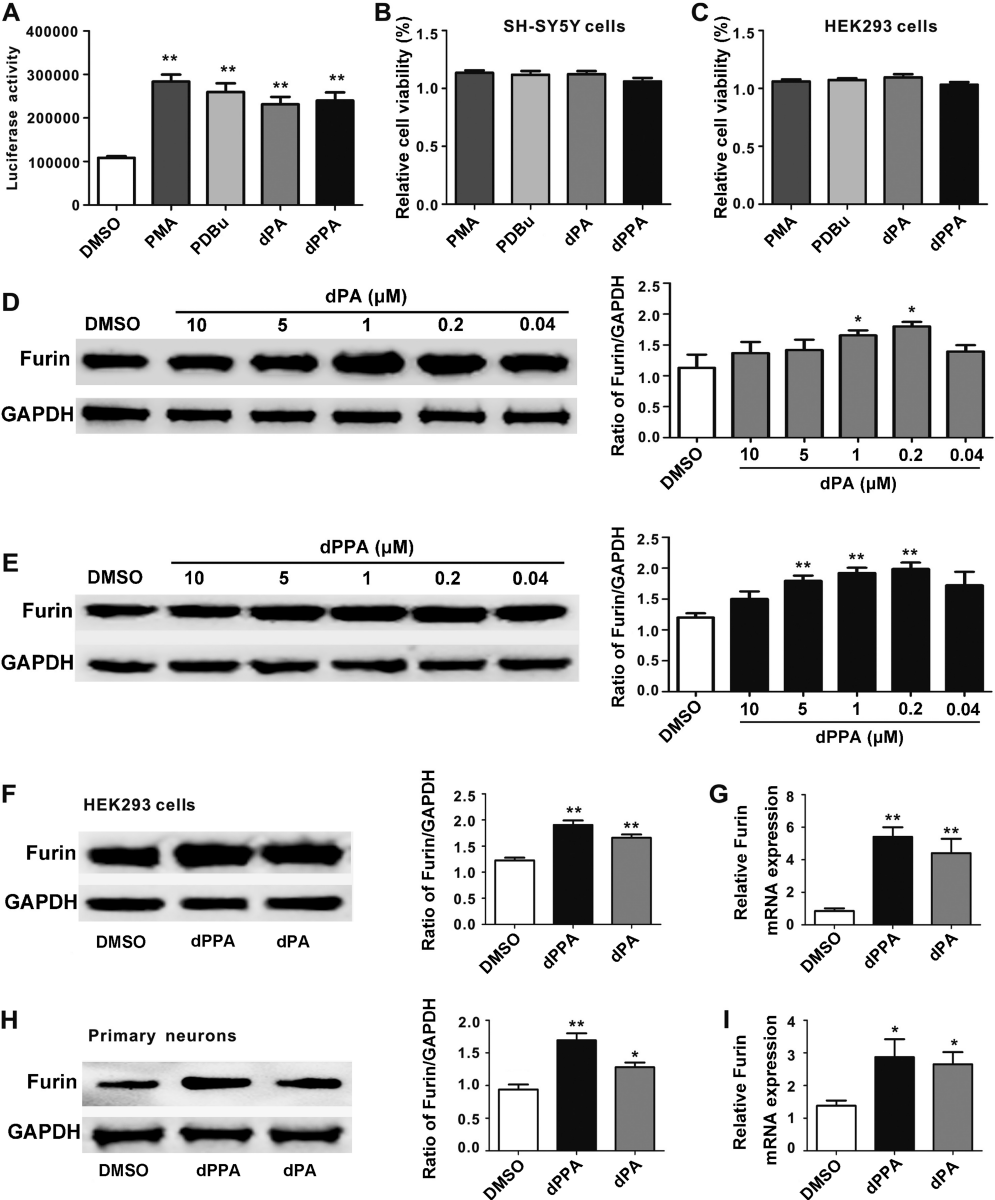
dPA/dPPA elevates furin expression (**A**) SH-SY5Y cells stably expressing *furin* P1 were treated for 24 h with 10 μM PMA, PDBu, dPA and dPPA that were found from 6988 kinds of traditional Chinese Medicine using high-throughput screening. They all promote luciferase activity of *furin* P1 promoter (**P < 0.01). **(B** and **C**) SH-SY5Y and HEK293 cells were treated with 10 μM PMA, PDBu, dPA and dPPA for 72 h and cell viability was assessed by CCK-8 assay. (**D** and **E**) SH-SY5Y cells were treated with dPA (D) and dPPA (E) at different concentrations (0.04–10 μM) for 72 h, and the expression of furin was determined by Western blot analysis (*P < 0.05, **P < 0.01, compared to DMSO group). (**F** and **H**) HEK293 cells or primary neurons were treated with 0.2 μM dPA and dPPA for 72 h, and the representative Western blotting images show that the expression of furin is significantly increased compared with control (*P < 0.05, **P < 0.01). (**G** and **I**) Cells were treated as described in Figure F and H, the mRNA level of *furin* was determined by real-time PCR. *P < 0.05, **P < 0.01. PMA, phorbol 12-myristate 13-acetate; PDBu, phorbol (12, 13)-dibutyrate; dPA, 12-deoxyphorbol 13-acetate; dPPA, 12-deoxyphorbol 13-phenylacetate 20-acetate.

SH-SY5Y cells stably expressing *furin* P1 promoter were seeded onto 384-well plates (3000 cells per well) for 24 h [18], and were treated with 6990 small molecules provided by the Chinese National Academy (Shanghai, China) at a concentration of 10 μM for 24 h. Luciferase assay revealed that the four phorbol esters PMA (phorbol 12-myristate 13-acetate), PDBu (phorbol 12, 13-dibutyrate), dPA (12-deoxyphorbol 13-acetate) and dPPA (12-deoxyphorbol 13-phenylacetate 20-acetate) significantly increased luciferase activity (Figure 1A, Supplementary Figure 1). 10 μM of these drugs did not interfere with the viability in both SH-SY5Y and HEK293 cells (Figure 1B and 1C). Since PMA and PDBu may induce carcinogenesis [19], we then selected dPA and dPPA that have been proved as antineoplastic agents [20, 21], for further study. We first assessed the effect of dPA or dPPA on furin protein expression in SH-SY5Y cells. Dose response analysis showed that the best concentration of dPA or dPPA for furin enhancement was 0.2 μM (Figure 1D and 1E), which was chosen throughout the study. In addition to SH-SY5Y cells, HEK293 cells also exhibited significantly increased furin protein and mRNA after dPA/dPPA treatment (Figure 1F and 1G). Similar results were found in rat primary cortical neurons (Figure 1H and 1I). These results indicated that dPA/dPPA effectively enhanced furin transcription in neuronal cells.

### Different effect of PKC inhibitors on dPPA/dPA regulation of furin expression

**Figure 2:**
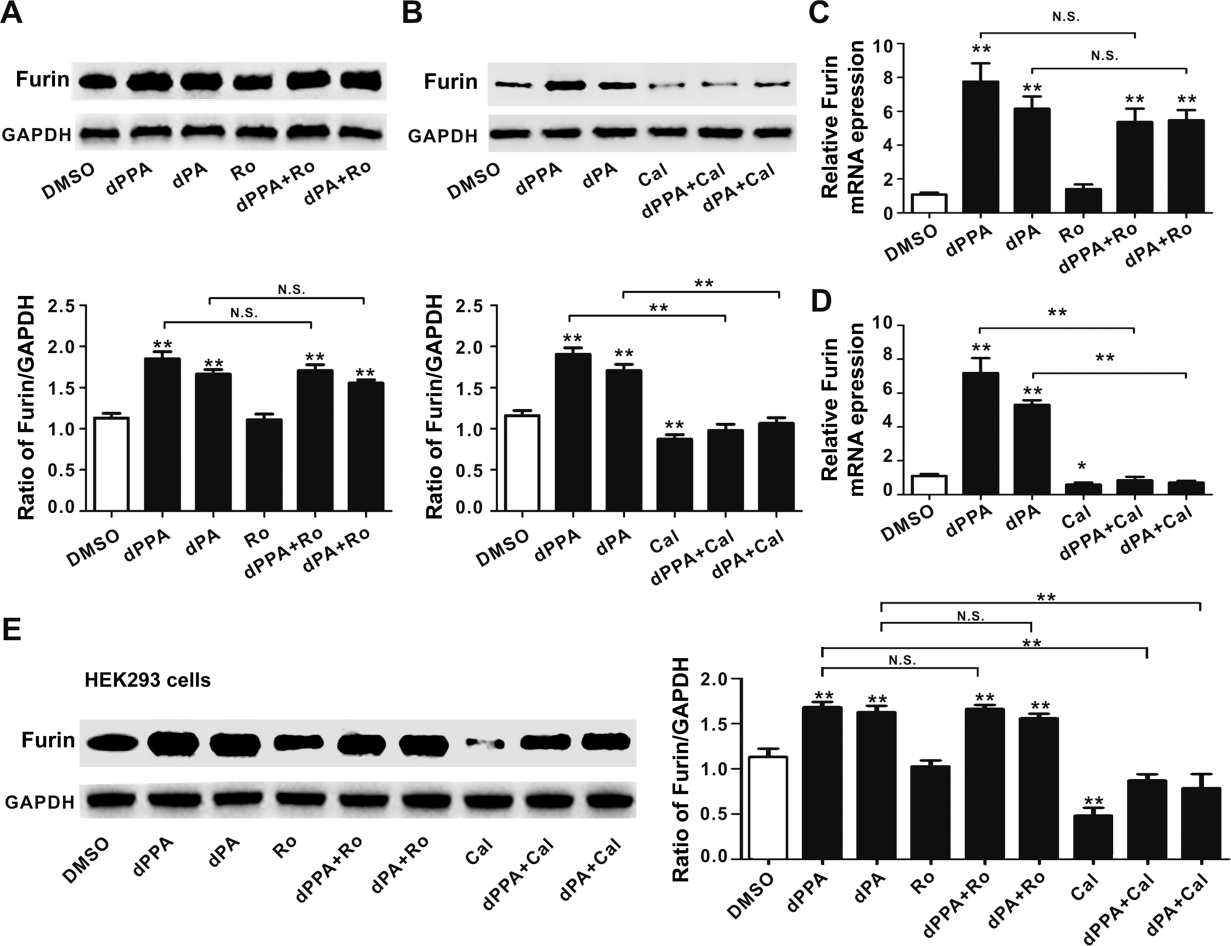
Effect of PKC inhibitors on dPA/dPPA induced expression of furin (**A**) SH-SY5Y cells were treated with 10 μM Ro318220 (Ro) in the absence or presence of 0.2 μM dPPA or dPA for 72 h, and the Western blotting results show that Ro318220 does not affect the up-regulation of furin induced by dPPA or dPA (**P < 0.01, n.s: non significant, compared to control). (**B**) SH-SY5Y cells were treated with 0.5 μM calphostin C (Cal) in the absence or presence of 0.2 μM dPPA or dPA for 72 h, and the Western blotting results show that the induction of furin by dPPA or dPA is diminished in the presence of calphostin C (**P < 0.01, compared to control). (**C** and **D**) SH-SY5Y cells were treated as described in Figure 2A and 2B, the mRNA level of *furin* was determined by real-time PCR (*P < 0.05, **P < 0.01, n.s: non significant, compared to control). (**E**) HEK293 cells were treated with 10 μM Ro318220 (Ro) or 0.5 μM calphostin C (Cal) in the absence or presence of 0.2 μM dPPA or dPA for 72 h, and the Western blotting results show that calphostin C rather than Ro318220 inhibits the up-regulation of furin induced by dPPA or dPA. ** *P* < 0.01, n.s: nonsignificant, compared to control.

Phorbol esters are known to be PKC activators [22, 23]. To test whether PKC may be involved in furin expression, we first assessed the effect of Ro318220 (a PKC inhibitor), which competes with PKC for ATP binding [19, 24]. SH-SY5Y cells were treated with 10 μM Ro318220 in the absence or presence of 0.2 μM dPPA or dPA for 72 h. Figure 2A showed that Ro318220 alone had no effect on furin expression compared to control, and the inhibition of PKC by Ro318220 did not affect the up-regulation of furin induced by dPPA or dPA. Next, we tested the effect of another PKC inhibitor calphostin C that competitively inhibits phorbol ester binding to the C1 domain [19, 25]. We found that 0.5 μM calphostin C alone significantly reduced the basal furin protein level compared to control. In the presence of calphostin C, the induction of furin by dPPA or dPA was diminished (Figure 2B, *P* < 0.01).

To further validate the results of Western blotting, we investigated whether mRNA levels of *furin* were analogously affected by Ro318220 or calphostin C. Similarly, Ro318220 did not affect the basal level and dPPA/dPA-induced upregulation of *furin* mRNA in SH-SY5Y cells (Figure 2C). In contrast, calphostin C significantly reduced the basal level of *furin* mRNA and blocked the induction of *furin* mRNA by dPPA or dPA in SH-SY5Y cells (Figure 2D). In addition, as expected, calphostin C rather than Ro318220 inhibited the up-regulation of furin protein induced by dPPA or dPA in HEK293 cells (Figure 2E). These results suggested that PKC C1 domain, rather than the kinase activity, was involved in dPPA/dPA regulation of furin expression.

### Nucleotides −7925 to −7426 were sufficient to mediate dPPA/dPA enhancement of *furin*

**Figure 3:**
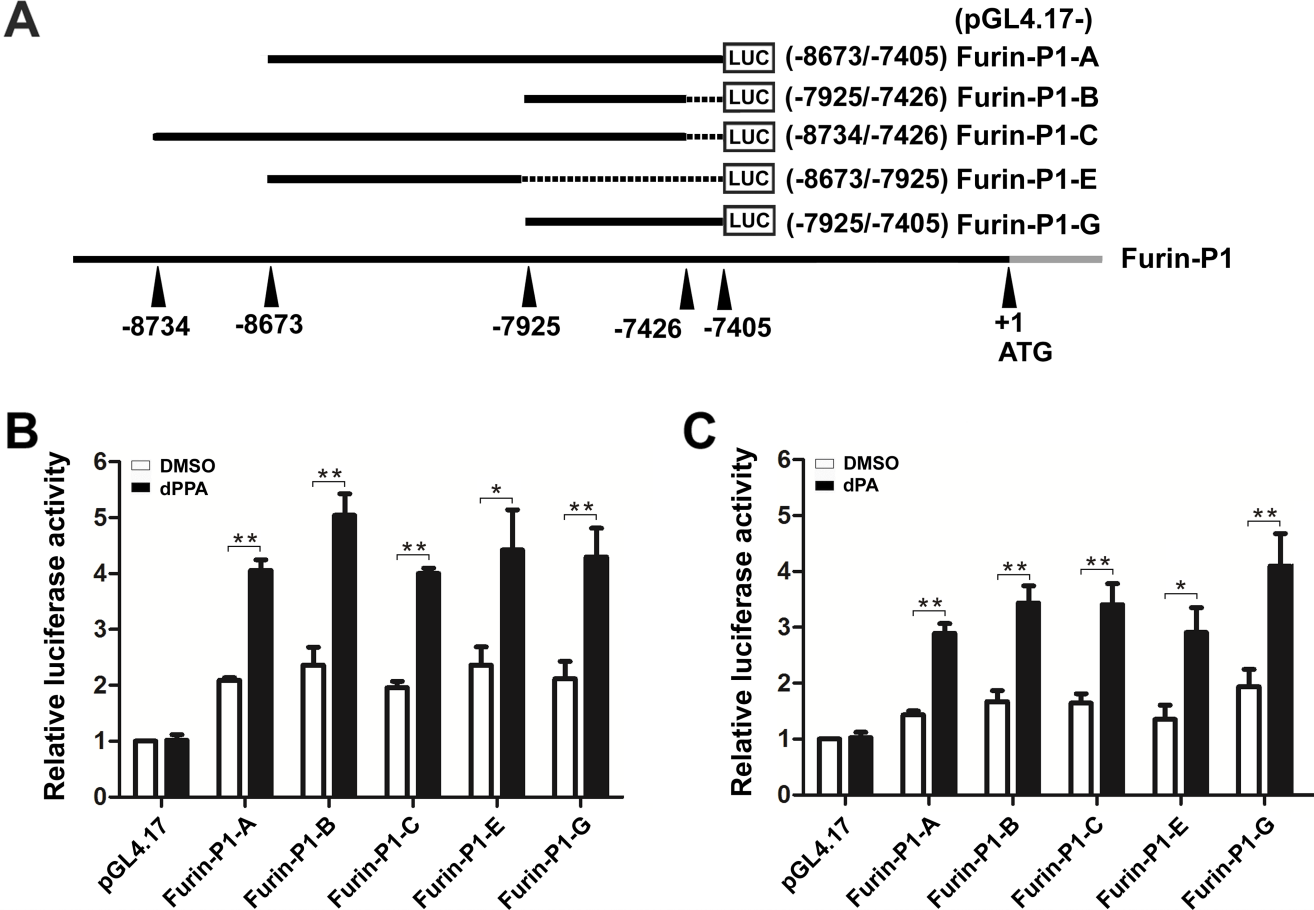
Identification of the core fragments in furin P1 promoter (**A**) Schematic representation of the truncated *furin* P1 promoter fragments. Numbers indicate the relative positions with respect to the ATG start codon (+1). The black line represents the 5′ genomic region of the furin gene, the gray line represents the furin cDNA sequence, and the rectangle represents the firefly luciferase coding region (LUC). (**B** and **C**) SH-SY5Y cells were transiently transfected with Furin-P1-A, Furin-P1-B, Furin-P1-C, Furin-P1-E, Furin-P1-G and pGL4.17 (negative control) for 24 h and then treated with 0.2 μM dPPA (B) or dPA (C) for another 24 h. The luciferase activity was measured and the relative fold activation of each truncated promoter fragments normalized to pGL4.17 internal standard (the luciferase activity of pGL4.17 treated with DMSO was set as 1) was presented. The results represent the average and SEM of three independent experiments. *P < 0.05, **P < 0.01, compared to control.

To further identify the core elements that are responsible for dPPA/dPA regulation of furin, the different sequentially deleted 5′-flanking regions of *furin* P1 promoter (pGL4.17-furin-P1-A, pGL4.17-furin-P1-B, pGL4.17-furin-P1-C, pGL4.17-furin-P1-E and pGL4.17-furin-P1-G) were generated (Figure 3A). SH-SY5Y cells were transiently transfected with each truncated promoter for 24 h and then were treated with 0.2 μM dPPA or dPA for 24 h. We found that except P1-E, all promoter fragments responded well to dPPA, all of which contained P1-B fragment sequence (nucleotides −7925 to −7426, Figure 3B). Similarly, in dPA treated cells, P1-B luciferase activity was significantly increased (Figure 3C). These results indicated that nucleotides −7925 to −7426 in *furin* P1 promoter were sufficient to mediate dPA/dPPA-induced expression of furin.

### dPPA/dPA regulation of furin involved transcription factor CEBPβ

**Figure 4:**
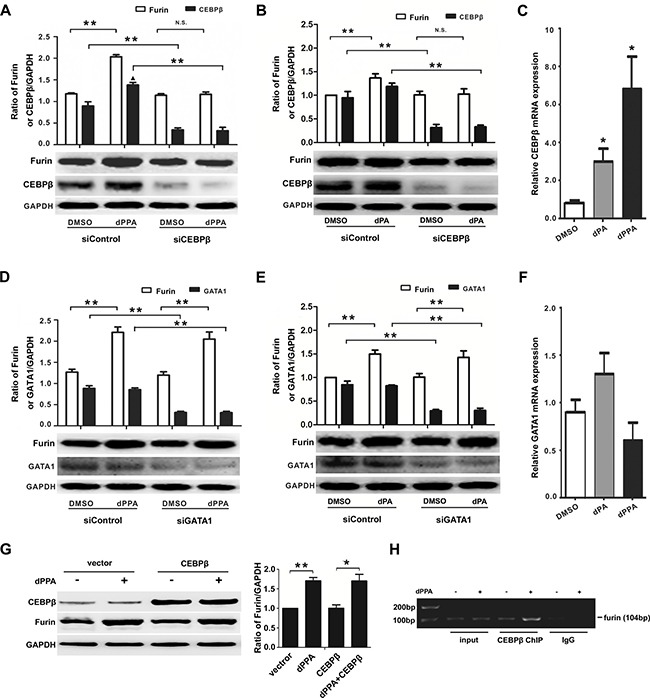
dPPA/dPA regulation of furin requires transcription factor CEBPβ (**A** and **B**) SH-SY5Y cells were transfected with *CEBPβ* siRNA (siCEBPβ) or control siRNA (siControl) for 24 h and then treated with 0.2 μM dPPA (A) or dPA (B) for 72 h. Western blotting results show that the inhibition of *CEBPβ* blocks the effect of dPPA/dPA on furin expression. (**C**) SH-SY5Y cells were treated with 0.2 μM dPPA or dPA for 72 h, and mRNA levels of *CEBPβ* were determined by real-time PCR. (**D** and **E**) SH-SY5Y cells were transfected with *GATA1* siRNA (siGATA1) or control siRNA (siControl) for 24 h and then treated with 0.2 μM dPPA (D) or dPA (E) for 72 h. Western blotting results show that dPPA/dPA still increases the expression of furin after *GATA1* is knockdown. (**F**) SH-SY5Y cells were treated with 0.2 μM dPPA or dPA for 72 h, and mRNA levels of *GATA1* were determined by real-time PCR. (**G**) SH-SY5Y cells were transfected with pcDNA3-CEBPβ or pcDNA3 (vector) for 24 h and then treated with 0.2 μM dPPA for 72 h, and the Western blotting results show that CEBPβ overexpression did not affect the basal furin protein level and the effect of dPPA. (**H**) SH-SY5Y cells were treated with 0.2 μM dPPA for 72 h, and then ChIP analysis was performed using ChIP assay kits with a CEBPβ antibody. The input or immunoprecipitated DNA was subjected to PCR amplification using primers specific to the *furin* promoter. *P < 0.05, **P < 0.01, n.s: nonsignificant, compared to control.

It is reported that *furin* P1 promoter has GATA1 element and can be trans-activated by transcription factor CEBPβ (CCAAT/enhancer-binding protein β) [26, 27]. To test whether direct manipulation of *CEBPβ* or *GATA1* may affect furin expression induced by dPPA, we assessed the effect of dPPA/dPA in SH-SY5Y cells transiently transfected with *CEBPβ* or *GATA1* siRNA. As shown in Figure 4A and 4B, *CEBPβ* siRNA led to dramatically decreased CEBPβ protein expression. CEBPβ knockdown did not reduce the basal level of furin protein but diminished dPPA/dPA-induced enhancement of furin in SH-SY5Y cells (Figure 4A and 4B). Interestingly, dPA/dPPA seemed to increase CEBPβ transcription in SH-SY5Y cells, as revealed by qPCR (Figure 4C). In *GATA1* siRNA transfected cells, the basal level of furin was not altered as compared to control. Knockdown of GATA1 did not prevent dPPA/dPA-induced enhancement of furin in SH-SY5Y cells (Figure 4D and 4E). The transcription of *GATA1* was also not affected by dPPA/dPA treatment (Figure 4F). Next, we assessed the effect of dPPA on furin expression in cells transiently overexpressing CEBPβ. However, overexpression of CEBPβ did not affect the basal furin protein level and the effect of dPPA (Figure 4G). A ChIP assay further revealed that CEBPβ was able to bind to the *furin* promoter, and this binding was increased in the presence of dPPA (Figure 4H). These results indicated that CEBPβ responsive element was involved in dPPA/dPA regulation of furin expression.

### ERK and PI3K signaling were involved in dPPA regulation of furin

**Figure 5:**
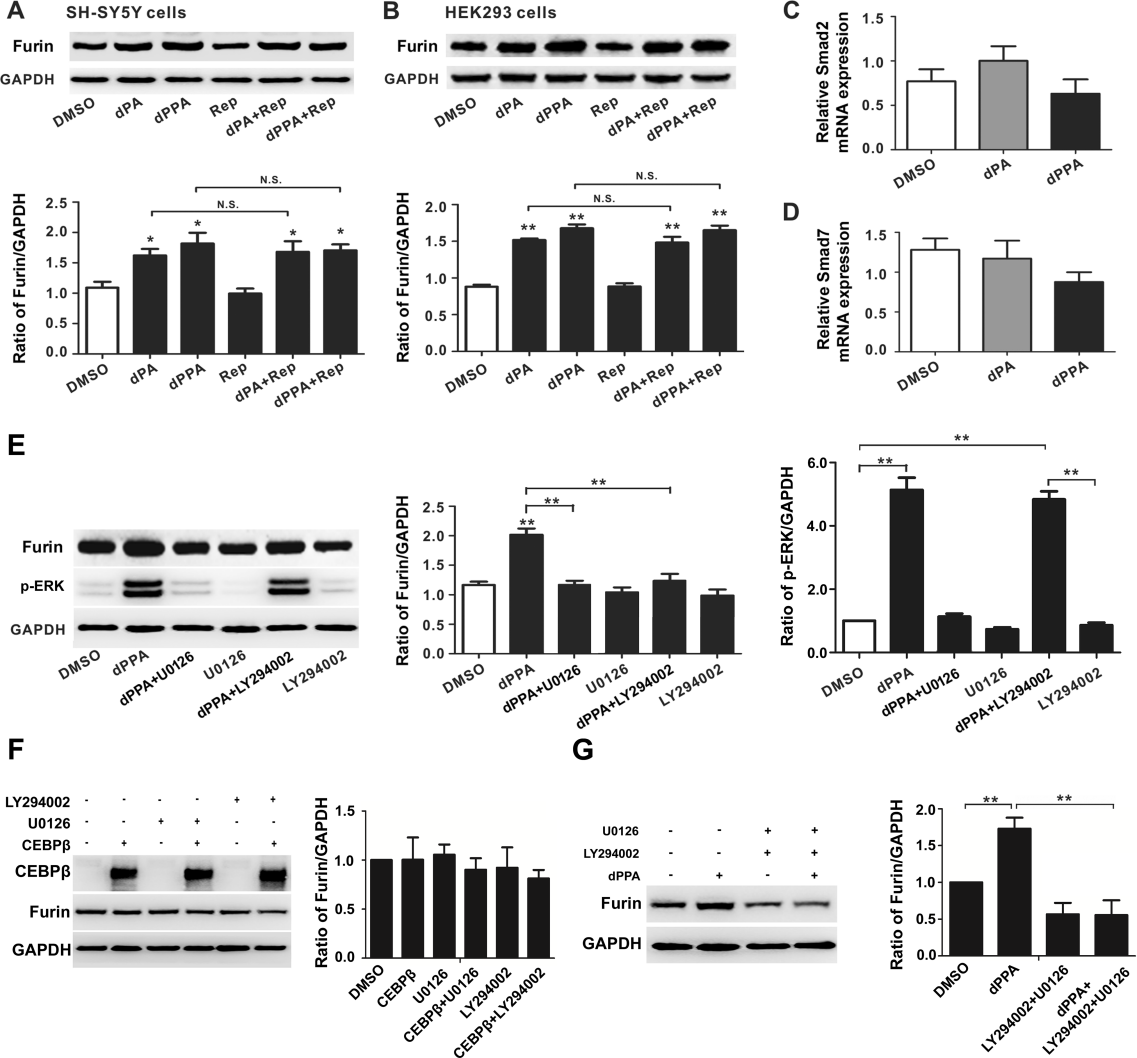
ERK or PI3K rather than TGFβ signaling is involved in dPPA induced furin expression (**A** and **B**) SH-SY5Y (A) and HEK293 cells (B) were treated with 25 μM RepSox (Rep, TGFβ receptor inhibitor) in absence or presence of dPA or dPPA for 72 h, and the Western blotting results show that dPA or dPPA increases the expression in the presence of RepSox. (**C** and **D**) SH-SY5Y cells were treated with 0.2 μM dPPA or dPA for 72 h, and mRNA levels of *Smad2* and *Smad7* were determined by real-time PCR. (**E**) SH-SY5Y cells were treated with 20 μM U0126 (ERK inhibitor) or 10 μM LY294002 (PI3K inhibitor) in absence or presence of 0.2 μM dPPA for 72 h, and the Western blotting results show that inhibition of ERK or PI3K pathway prevents dPPA induced furin expression. (**F**) SH-SY5Y cells were transfected with pcDNA3-CEBPβ or pcDNA3 (vector) for 24 h and then treated with 20 μM U0126 or 10 μM LY294002 for 24 h, and the Western blotting results show that in cells overexpressing CEBPβ, U0126 or LY294002 had no effect on furin expression (**G**) SH-SY5Y cells were treated jointly with 20 μM U0126 and 10 μM LY294002 in absence or presence of 0.2 μM dPPA for 72 h, and the Western blotting results show that inhibition of ERK and PI3K completely blocks dPPA induced furin expression. *P < 0.05, **P < 0.01, n.s: nonsignificant, compared to control.

Previous study has demonstrated that TGFβ stimulates P1 promoter activity, which involves smad2 (mothers against DPP homolog 2) and smad7 [28]. To test whether TGFβ may be involved dPA/dPPA regulation of furin, SH-SY5Y and HEK293 cells were treated with 25 μM RepSox (TGFβ pathway inhibitor) in absence or presence of dPA or dPPA for 72 h. We found that RepSox did not cause significant alteration of furin protein compared to control. The expression of furin remained significantly increased after dPA or dPPA in the presence of RepSox (Figure 5A and 5B). dPA or dPPA also failed to increase the mRNA levels of *smad2* and *smad7* in SH-SY5Y cells (Figure 5C and 5D). These results indicated TGFβ signaling was not involved in dPA/dPPA augmentation of furin.

ERK (extracellular regulated protein kinases) signaling is known to play important role in PKC-regulated cellular function [29]. Interestingly, CEBPβ activity can be regulated by ERK [30, 31]. To test whether ERK may be involved in dPPA regulation of furin, we assessed the effect of U0126 (ERK inhibitor) in SH-SY5Y cells, LY294002 (PI3K inhibitor) was used as a control. As expected, U0126 or LY294002 diminished rather than completely blocked the effect of dPPA on furin protein expression (Figure 5E). Meanwhile, in cells overexpressing CEBPβ, U0126 or LY294002 had no effect on furin expression (Figure 5F). The combined treatment of U0126 with LY294002 completely inhibited dPPA induced furin expression (Figure 5G). These results suggested that ERK and PI3K signaling pathways were involved in dPPA regulation of furin.

## DISCUSSION

Phorbol ester regulation of neuronal gene expression has been documented. In GT1 cell line, phorbol esters enhance gonadotropin-releasing hormone and *c-fos* genes [32, 33]. In rat hypothalamic culture, 12-O-tetradecanoylphorbol-13-acetate increases the protein level of thyrotropin releasing hormone and CREB [34]. In neuroblastoma cell lines, phorbol ester regulates vasoactive intestinal peptide gene expression [35]. Here we provide evidence that phorbol esters significantly increase *furin* gene expression in neuronal cells. We also show that this effect is not neuron-specific, as similar effect and mechanisms have been shown in HEK293 cells as well.

Phorbol esters enter freely into the cells and are preferentially localized to membraneous structures including endoplasmic reticulum, Golgi, mitochondrial and nuclear membranes [36, 37]. Protein kinase C (PKC) isoforms have been identified as phorbol ester receptors [22, 38], which play important roles in various biological phenomena [39, 40]. It seems that the C1 domain mediates phorbol ester binding to PKC [41, 42]. Phorbol esters insert into the hydrophilic cleft of C1 domains, favoring PKC association with lipid membranes [43, 44]. The conformational flexibility of C1 domains determines differential activation mechanisms of PKCs [45]. In our studies, dPA/dPPA-induced furin expression is blocked by calphostin C that competitively binds to C1 domain of PKC, but not by Ro318220 that acts on the catalytic domain of PKC by competing with ATP binding, suggesting that the C1 domain rather than the catalytic domain of PKC mediates the up-regulation of *furin* gene.

*Furin* gene is controlled by three distinct promoters known as P1, P1A and P1B [26]. While P1A and P1B promoters are considered as housekeeper genes, P1 promoter is under the control of variety of transcriptional factors, including CEBPβ, GATA1, HIF-1, Smads, CREB and CDX2 [26–28, 46–48]. In SH-SY5Y cells in our study, nucleotides −7925 to −7405 in P1 promoter effectively mediate dPPA or dPA induced luciferase activity. We further identify that CEBPβ is involved in dPPA/dPA induced enhancement of furin, as revealed by siRNA experiments. However, another transcription factor GATA1 was not involved in dPPA/dPA regulation of *furin* gene in SH-SY5Y cells, which is in contrast to the previous report that in megakaryocytes, PMA promotes furin expression through GATA1 in P1 promoter [27]. As described above, PMA and dPPA may differ in functions. The former is carcinogenic while the latter is antineoplastic [19–21]. Another possibility is that P1 promoter may be activated in a tissue- and cell differentiation-dependent manner [26]. It is also unlikely that TGFβ signaling is involved, as TGFβ receptor antagonist does not prevent dPPA effect on furin. In HepG2 cells, the key fragments for TGFβ and Smads to take effect are at position −8734 and −7925 [49], which are not included in the region (−7925 to −7405) identified in our study.

C1 domain has been considered as phorbol ester receptor in PKC and other proteins [50]. Interestingly, many of these proteins including PKD (protein kinase D), c-Raf (Raf-1 proto-oncogene, serine/threonine kinase) and Ras-GRP (Ras guanyl releasing protein) share with PKC the common signaling pathway, resulting in activation of Raf-ERK [29, 50, 51]. It is reported that ERK could promote the phosphorylation of CEBPβ [30, 31]. Thus CEBPβ may link phorbol ester to furin expression. However, why PI3K inhibitor also diminishes dPPA effect on furin is not well-understood. Study has demonstrated that promotion of neurite growth by phorbol esters can be blocked by PI3K inhibitor [52]. Genes associated with PI3K signaling are significantly affected by PMA in human monocytic cell line [53]. A recent study shows that PI3K is involved in EGF-induced prolactin receptor expression, which is dependent on Sp1/CEBPβ complex [54]. Thus, it may be reasonable to speculate that phorbol esters act on C1 domain proteins and plasma membrane receptors leading to downstream activation of ERK and PI3K. In our study, ERK and PI3K may work synergistically, as the basal level of furin protein was reduced only when both inhibitors are administered (Figure 5G).

It is interesting that both knockdown and overexpression of CEBPβ fail to affect basal furin protein (Figure 4). While CEBPβ knockdown prevents dPPA/dPA enhancement of furin (Figure 4A and 4B), CEBPβ overexpression is without effect (Figure 4G). We speculate that CEBPβ is constitutively active, and its functional role may be tightly regulated by phosphorylation, which involves other CEBP members. Study has shown that CEBPβ and CEBPδ are key transcriptions factors that mediate cytokine production in macrophage. In contrast, macrophages deficient of CEBPβ or CEBPδ fail to show significant decrease of cytokines [55]. The synergistic effect of CEBPβ and CEBPδ has been shown for mice lacking these two factors that exhibit defective adipocyte differentiation [56]. Although it is not currently clear which CEBP subtypes may play a role, they all contain phosphorylation sites [30]. Some of them are known to be regulated by PKC and downstream signaling resulting in altered DNA binding and gene expression [57, 58]. Thus, it is not surprising that overexpression of CEBPβ alone fails to induce furin expression (Figure 4G), which also fails to bypass the ERK or PI3K inhibition on furin induction by dPPA. A working model is that the constitutively active CEBPβ may interact with other CEBP members, and their bindings to P1 promoter are critically dependent on ERK and PI3K. In line with this, ERK phosphorylation of another transcription factor USF1 (upstream transcription factor 1) is suggested to be prerequisite to USF1-mediated gene expression [59, 60].

**Figure 6:**
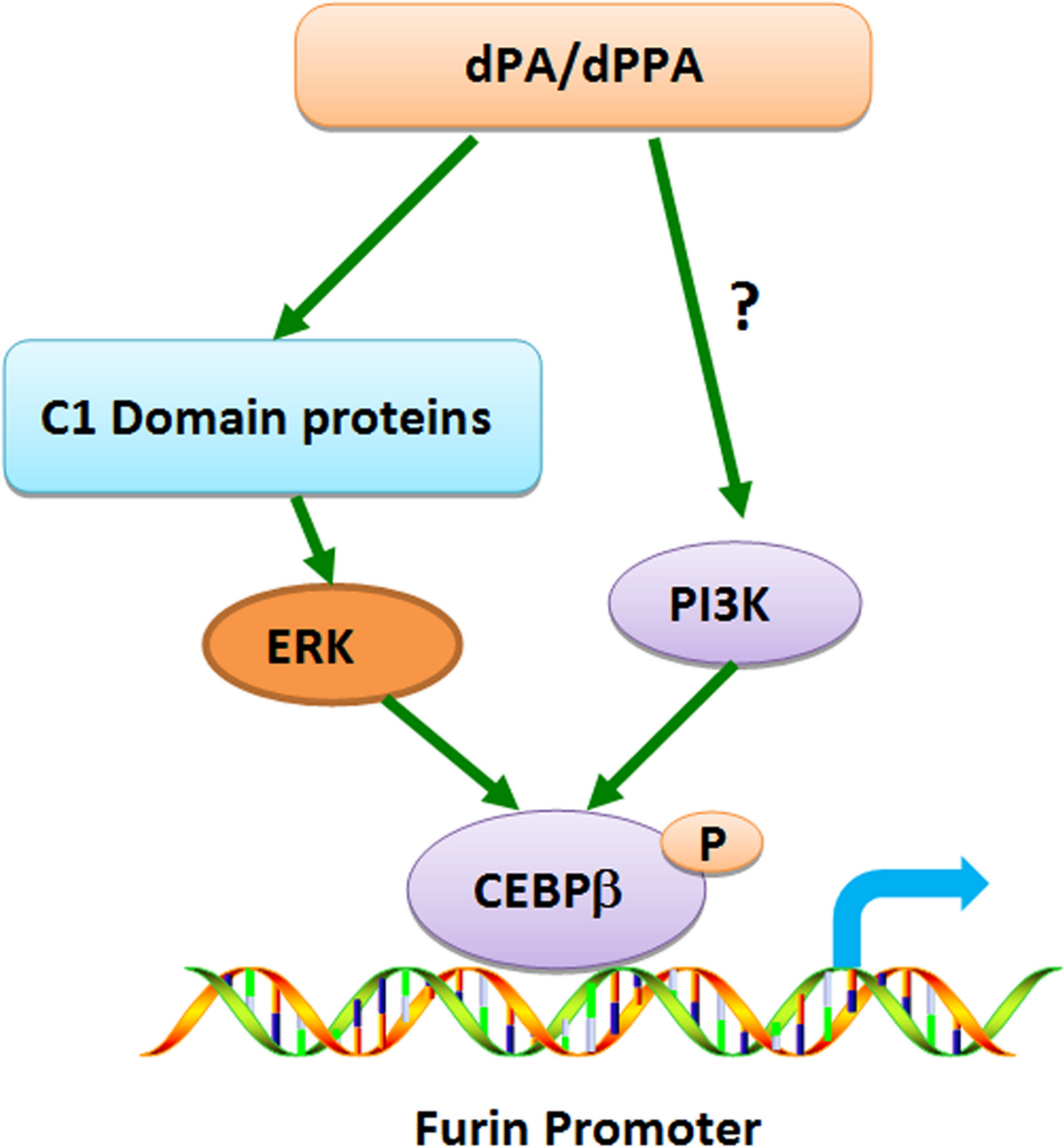
Schematic diagram depicting the possible mechanisms through which dPPA/dPA regulates furin transcription dPPA/dPA enters freely into the cells and preferentially binds C1 domain-containing proteins and perhaps plasma membrane receptors. This leads to the downstream activation of ERK and PI3K, which may synergistically promote CEBPβ association with P1 promoter and with other CEBP members (not shown), resulting in the enhanced expression of furin.

We propose the potential mechanisms that dPPA/dPA increases furin expression. Upon dPPA/dPA binding to C1 domain containing proteins and perhaps plasma membrane receptors, downstream ERK and PI3K are activated, which may work synergistically to promote CEBPβ association with P1 promoter and perhaps with other CEBP members, leading to the increased expression of furin protein (Figure 6). However, how CEBPβ may be associated with other CEBP members, and which subtypes may be involved, remain to be investigated in the future. Nonetheless, dPPA/dPA regulation of furin in neuronal cells may shed new light on the understanding of neurological diseases, especially Alzheimer's disease.

## MATERIALS AND METHODS

### Chemicals

The PMA, PDBu, dPA, dPPA, LY294002 (PI3K inhibitor) and U0126 (ERK inhibitor) were obtained from Sigma (Saint Louis, USA). Calphostin C was ordered from Cayman (Michigan, USA). RepSox (TGFβ pathway inhibitor) was from MedChemExpress (MCE, New Jersey, USA). Ro318220 were from Santa Cruz (Santa Cruz Biotechnology, California, USA). All chemicals were dissolved in DMSO (sigma) to generate a 50 mM stock solution, with final dilutions of at least 1:2000.

### Plasmids construction

Human genomic DNA was extracted from cultured cells and used as a template for amplification of a 1268bp (−8673/−7405) fragment, named Furin-P1-A, consisting of the human *furin* P1 promoter and its upstream sequence [18]. The Furin-P1-A was further divided into four fragments which contain DNA fragments from −7925 to −7426bp (Furin-P1-B), −8734 to −7426bp (Furin-P1-C), −8673 to −7925bp (Furin-P1-E), and −7925 to −7405bp (Furin-P1-G), respectively. The fragments of *furin* P1 were subsequently subcloned into the luciferase reporter vector pGL4.17 (Promega, Madison, USA). All constructs were amplified by PCR with their specific primers listed in Table 1 and confirmed by DNA sequencing.

**Table 1:** Primers of different *furin* P1 promoters for plasmids construction

Name	Sequence	Size (bp) (Start-Stop)
Furin-P1-A	5′-GCCGGCTAGCACCACCATCCTGTGACGTTCCC-3′	1268
	5′-CCCAAGCTTCTGCTGCTTCCCCGCCACTC-3′	(−8673/−7405)
Furin-P1-B	5′-GCCGGCTAGCCTCCATGCAACCCTACTAGAGAGGTG-3′	499
	5′-CCCAAGCTTCTGCAGCTGCAACAGTCAGGC-3′	(−7925/−7426)
Furin-P1-C	5′-GCCGGCTAGCCTAGCTGTCTCAGAGCTTAGTTCCCAG-3′	1308
	5′-CCCAAGCTTCTGCAGCTGCAACAGTCAGGC-3′	(−8734/−7426)
Furin-P1-E	5′-GCCGGCTAGCACCACCATCCTGTGACGTTCCC-3′	748
	5′-CCCAAGCTTGGAGGTACCTGGGACACACCAGG-3′	(−8673/−7925)
Furin-P1-G	5′-GCCGGCTAGCCTCCATGCAACCCTACTAGAGAGGTG-3′	520
	5′-CCCAAGCTTCTGCTGCTTCCCCGCCACTC-3′	(−7925/−7405)

### Cell culture, stable cell line generation and pharmacological treatments

Human neuroblastoma cells (SH-SY5Y) and human embryonic kidney 293 cells (HEK293) were obtained Shanghai Institute of Biological Sciences (Chinese Academy of Sciences, China). SH-SY5Y cells were maintained in Dulbecco's modified eagle's medium/nutrient mixture F12 (DMEM/F12, Gibco, Grand Island, USA) with 10% FBS (Gibco) plus 100 U/ml penicillin and 100 μg/ml streptomycin. HEK293 cells were grown in DMEM (Gibco), supplemented with 10% FBS, 100 U/ml penicillin and 100 μg/ml streptomycin.

SH-SY5Y cells stably expressing *furin* P1 promoters were generated by using G418 (0.5 mg/ml) selection to screen for SH-SY5Y clones transiently transfected with pGL4.17-Furin-P1 using lipofectamine 2000 (Invitrogen, Carlsbad, USA). The medium was replaced by complete medium after 6 h, and cells were incubated for 24 h. The cell monolayers were then grown in DMEM/F12 with 10% FBS and 0.2 mg/ml G418 for later screening of cells.

Primary cortical neurons were prepared from Sprague-Dawley rat embryos at day 17–18 and treated with 0.25% trypsin-EDTA for 15 min. The digested tissues were dissociated by trituration and plated on dishes overnight, and the cultures were maintained in DMEM for 24 h. Subsequently, replaced the medium with fresh Neurobasal medium (Invitrogen) supplemented with 2% B27 (Invitrogen), 100 U/ml penicillin, 100 μg/ml streptomycin and 0.5 mM glutamine. Every 2–3 days, half of the medium was removed and replenished with the fresh Neurobasal medium. Neurons were used for experiments at 7–11 days *in vitro*. All procedures were carried out in accordance with the Chongqing Medical University guidelines for the care and use of laboratory animals. All cells were maintained at 37°C under 5% CO_2_ atmosphere.

Cells were transfected with luciferase reporter plasmids or pcDNA3-CEBPβ (Origene, Rockville, USA) and then were treated with PMA, PDBu, dPA or dPPA with indicated concentrations for 72 h. To investigate the signaling pathways involved in the dPPA/dPA-mediated effect, cells were treated with the 0.5 μM calphostin C (PKC inhibitor), 10 μM Ro318220 (PKC inhibitor), 25 μM RepSox (TGFβ pathway inhibitor), 10 μM LY294002 (PI3K inhibitor) or/and 20 μM U0126 (ERK inhibitor) for 72 h in the absence or presence of dPPA/dPA.

### Luciferase activity assay

For high-throughput small-molecule screening, SH-SY5Y cells stably expressing *furin* P1 promoters were seeded onto 384-well plates for 24 h and were treated with 6990 small molecules at a concentration of 10 μM for 24 h. For identifying the core fragments in *furin* P1 promoter, SH-SY5Y cells were seeded into 96-well plates (0.8–1.0 × 10^4^ cells per well) 24 h before transfection. Then, cells were transiently transfected with 0.2 μg luciferase reporter plasmids (pGL4.17-Furin-P1-A∼E) and pGL4.17 (a negative control) using Lipofectamine 2000 according to the manufacturer's instructions. 24 hours after transfection, PMA, PDBu, dPA or dPPA (0.2 μM) was added to cells, and DMSO was used as a negative control. The luciferase activities were measured using a luciferase assay kit (Promega, Madison, Wisconsin, USA) according to the manufacturer's instructions and the relative fold activation of each construct normalized to pGL4.17 internal standard (the luciferase activity of pGL4.17 treated with DMSO was set as 1.0) was presented. The data presented as mean luciferase activity ± SEM.

### RNA interference

The siRNA oligonucleotides for human *CEBPβ* and *GATA1* were synthesized by Shanghai GenePharma Co., Ltd (Shanghai, China), and the non-targeting control siRNA (siControl) was used as the negative control. The *CEBPβ* siRNA sequence is 5′-GCUGACAGUUACACGUGGGtt-3′, *GATA1* siRNA sequence is 5′-GGUACUCAGUGCACCAACUtt-3′ and siControl sequence is 5′-UUCUCCGAAC GUGUCACGUtt-3′. SH-SY5Y cells were transfected with 50 nM siRNAs for 6 h using Lipofectamine 2000 according to the manufacturer's protocol. 24 h after transfection, the cells were treated with 0.2 μM dPPA for another 72 h. Inhibition of target genes was confirmed by Western blotting.

### RNA isolation and quantitative real-time PCR

Total RNA from cells with or without treated with chemicals was extracted with TRIzol reagent (TaKaRa, Dalian, China). cDNA synthesis was performed using a PrimeScript RT reagent kit (Vazyme Biotech, Nanjing, China). Quantitative real-time PCR (qPCR) reactions were performed on a Bio-Rad IQ^™^5 detection system (Bio-Rad, Hercules, CA, USA) with a SYBR green master mix (TaKaRa) as recommended by the manufacturer. The qPCR primers used in this study are listed in Table 2. Each sample was carried out in triplicate, and the average cycle threshold value (Ct) for housekeeping gene *GAPDH* was used to normalize the raw cycle threshold data. The relative mRNA expression levels of the individual samples were calculated by the 2^−ΔΔCT^ method.

**Table 2:** qPCR primers used in this study

Gene	Sequence	Tm
Furin	5′-TGCCACGCCTCATGTGCC-3′	64.00
	5′-GCTCTGGCTTTGCCGGGA-3′	63.30
Smad2	5′-GAGATATGGCTGGCACCCTG-3′	60.25
	5′-TGCCTTCGGTATTCTGCTCC-3′	59.82
Smad7	5′-GTCAAGAGGCTGTGTTGCTG-3′	59.41
	5′-ATCTGGACAGTCAGTTGGTTTG-3′	58.52
CEBPα	5′-GGAGCTGACCAGTGACAATGACC-3′	59.59
	5′-CTGGCAGCTGGCGGAAGAT-3′	58.02
CEBPβ	5′-GCCACGGCCACGGACACCTT-3′	63.44
	5′-TCGGCCGGCTTCTTGCAGTTCTT-3′	63.46
GATA1	5′-GTAGCGGGAATTGTGGGGAGGTG-3′	61.50
	5′-GGGAAAGGCATGAGGTGGCTAACA-3′	61.41

### Protein extraction and Western blot

All cells with or without treated with chemicals were lysed in RIPA buffer (50 mM Tris, 1 mM EDTA, 150 mM NaCl, 1% Triton X-100, 0.1% SDS, 0.5% sodium deoxylcholate) including protease inhibitors (Roche, Indianapolis, USA). Protein concentrations were measured using a BCA Protein Assay Kit (Dingguo, Beijing, China). Equal amounts of protein extracts were separated on a 12% SDS-PAGE gel and then transferred onto PVDF membrane (Millipore, Billerica, MA, USA). Membranes were blocked by 5% nonfat dry milk in TBST (25 mM Tris, pH 7.4, 1.5 M NaCl, and 0.05% Tween-20) for 1 h at RT and probed with primary antibodies against furin (Abcam, Cambridge, UK), CEBPβ (Cell Signaling Technology, Danvers, MA, USA), GATA1 (Cell Signaling Technology), and GAPDH (Proteintech, Wuhan, Chian) overnight at 4°C. The blots were washed and incubated for 1 h with HRP-conjugated anti-rabbit or anti-mouse secondary antibodies (Proteintech). The bands were visualized using an ECL reagent (Advansta Inc., Menlo Park, CA, USA) and a Fusion FX5 image analysis system (Vilber Lourmat, Marne-la-Vallée, France). Relative protein expression levels were calculated using the Quantity One software (Bio-Rad) with normalization to the GAPDH signal.

### Chromatin immunoprecipitation (ChIP)

SH-SY5Y cells were treated with 0.2 μM dPPA for 72 h, and then, ChIP analysis was performed using ChIP assay kits (Beyotime) according to the manufacturer's recommendations with a CEBPβ antibody (Santa Cruz Biotechnology, sc-9314) and control IgG (Abcam). The input control DNA or immunoprecipitated DNA was then subjected to PCR amplification using primers specific to the *furin* promoter (forward primer 5′-ACCAGAGCCCAGCGTTCAGCAG-3′ and reverse primer 5′-CATGGCCAGCCAGTGCTGCCA-3′). The PCR products were separated by 2% agarose gel electrophoresis and visualized with ethidium bromide staining.

### Cell viability assay

Cell viability was assessed using a CCK-8 Cell Counting Kit (Vazyme). Briefly, cells were seeded into 96-well plates (0.8–1.0 × 10^4^ cells per well) overnight and were then treated with DMSO (Control), PMA, PDBu, dPA or dPPA (10 μM) for 72 h. 10 μL of the CCK-8 solution was then added to each well and absorbance was measured at 450 nm with a microplate reader (BioTek, Winooski, Vermont, USA) after incubation for another 4 h. Each sample was carried out in triplicate, and the average optical density (OD) was used for calculation.

### Statistical analysis

All data were shown as mean ± SEM. Statistical analyses were performed with the Graphpad Prism software. Data were analyzed either by Student's unpaired *t*-test or one-way ANOVA followed by post hoc analysis. Differences were considered to be significant when *P* < 0.05.

## SUPPLEMENTARY MATERIALS FIGURE



## Abbreviations

AD: Alzheimer disease
ADAM10: A disintegrin and metalloproteinase domain-containing protein 10
BDNF: Brain derived neurotrophic factor
CEBPβ: CCAAT/enhancer-binding protein β
CNS: central nervous system
DNA: Deoxyribonucleic acid
dPA: 12-Deoxyphorbol 13-acetate
dPPA: 12-Deoxyphorbol 13-phenylacetate 20-acetate
ERK: extracellular regulated protein kinases
GATA1: GATA binding protein 1
NGF: Nerve growth factor
PCs: Proprotein convertases
PDBu: Phorbol 12, 13-dibutyrate
PI3K: Phosphoinositide 3-kinase
PKC: Protein kinase C
PMA: Phorbol 12-myristate 13-acetate
Smad2/7: mothers against DPP homolog2/7
TGFβ: Transforming Growth Factor β
