# Supplementary material for: Phorbol esters dPPA/dPA promote furin expression involving transcription factor CEBPβ in neuronal cells
Source: Oncotarget. 2017 Jun 19;8(36):60159–72. doi: 10.18632/oncotarget.18569 (PMC5601129; doi:10.18632/oncotarget.18569)
Supplement: Supplementary file 1 [file oncotarget-08-60159-s001.pdf]

Phorbol esters dPPA/dPA promote furin expression involving transcription factor CEBPβ in neuronal cells

Supplementary Materials

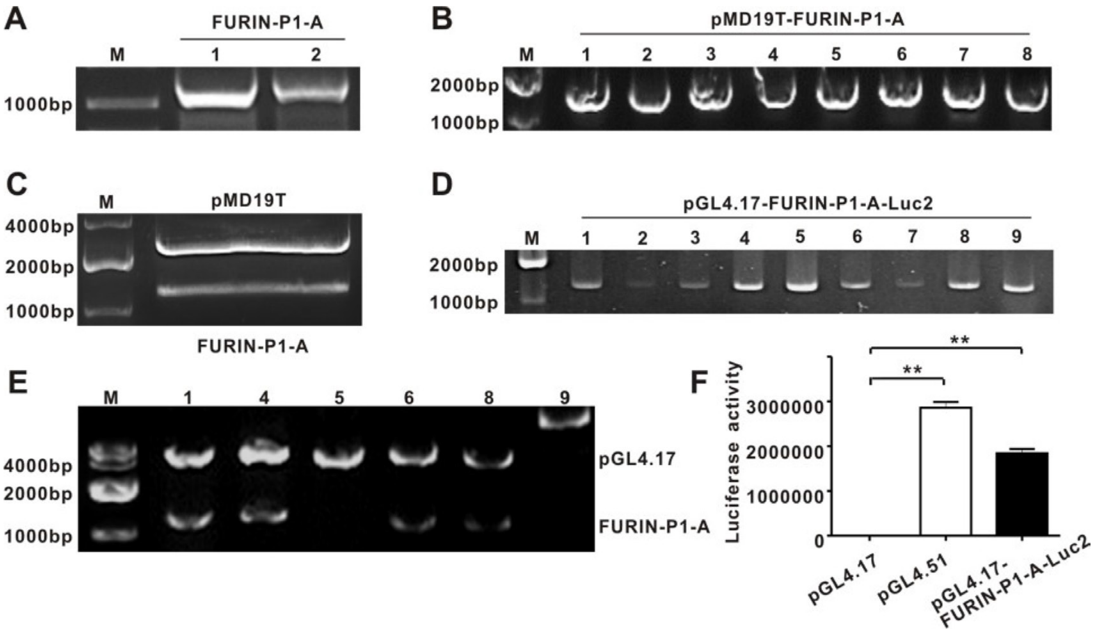

Supplementary Figure 1: Identification of recombination vector pGL4.17-FURIN-P1-A-Luc2. (**A**) Amplification of human *Furin* gene P1 promoter by PCR. M, DNA marker; Lane 1 and 2: Furin P1 promoter. (**B**) Identification of recombination vector pMD19T-FURIN-P1-A. M, DNA marker; Lane 1–9: The different PCR products. (**C**) Gel extraction of positive recombinants for sequencing. (**D**) Identification of recombination vector pGL4.17-FURIN-P1-A-Luc2. M, DNA marker; Lane 1–9: The different PCR products. (**E**) Identification of pGL4.17-FURIN-P1-A-Luc2 by restriction enzyme digestion. M, DNA marker; Lane 1–9: The different enzyme-digested products. (**F**) SH-SY5Y cells were transfected with pGL4.17-FURIN-P1-A-Luc2, pGL4.17 (negative control) and pGL4.51 (positive control) for 24 h. $^{**}P < 0.01$, compared to pGL4.17. Luciferase assays were performed with a GloMax 96 microplate luminometer.
